# Access to primary health care for immigrants: results of a patient survey conducted in 137 primary care practices in Ontario, Canada

**DOI:** 10.1186/1471-2296-13-128

**Published:** 2012-12-28

**Authors:** Elizabeth Muggah, Simone Dahrouge, William Hogg

**Affiliations:** 1 C.T. Lamont Primary Health Care Research Centre, Élisabeth Bruyère Research Institute, Ottawa, ON, Canada; 2 Department of Family Medicine, University of Ottawa, Ottawa, ON, Canada; 3 Institute of Population Health, University of Ottawa, Ottawa, ON, Canada; 4 43 Bruyere Street, Ottawa, ON, K1N 5C8, Canada

**Keywords:** Primary health care, Access to health care, Immigrants, Canada

## Abstract

**Background:**

Immigrants make up one fifth of the Canadian population and this number continues to grow. Adequate access to primary health care is important for this population but it is not clear if this is being achieved. This study explored patient reported access to primary health care of a population of immigrants in Ontario, Canada who were users of the primary care system and compared this with Canadian-born individuals; and by model of primary care practice.

**Methods:**

This study uses data from the Comparison of Models of Primary Care Study (COMP-PC), a mixed-methods, practice-based, cross-sectional study that collected information from patients and providers in 137 primary care practices across Ontario, Canada in 2005-2006. The practices were randomly sampled to ensure an equal number of practices in each of the four dominant primary care models at that time: Fee-For-Service, Community Health Centres, and the two main capitation models (Health Service Organization and Family Health Networks). Adult patients of participating practices were identified when they presented for an appointment and completed a survey in the waiting room. Three measures of access were used, all derived from the patient survey: First Contact Access, First Contact Utilization (both based on the Primary Care Assessment Tool) and number of self-reported visits to the practice in the past year.

**Results:**

Of the 5,269 patients who reported country of birth 1,099 (20.8%) were born outside of Canada. In adjusted analysis, recent immigrants (arrival in Canada within the past five years) and immigrants in Canada for more than 20 years were less likely to report good health compared to Canadian-born (Odds ratio 0.58, 95% CI 0.36,0.92 and 0.81, 95% CI 0.67,0.99). Overall, immigrants reported equal access to primary care services compared with Canadian-born. Within immigrant groups recently arrived immigrants had similar access scores to Canadian-born but reported 5.3 more primary care visits after adjusting for health status. Looking across models, recent immigrants in Fee-For-Service practices reported poorer access and fewer primary care visits compared to Canadian-born.

**Conclusions:**

Overall, immigrants who were users of the primary care system reported a similar level of access as Canadian-born individuals. While recent immigrants are in poorer health compared with Canadian-born they report adequate access to primary care. The differences in access for recently arrived immigrants, across primary care models suggests that organizational features of primary care may lead to inequity in access.

## Background

Immigrants comprise 20% of the Canadian population; a number that is increasing [1]. National surveys traditionally find a “healthy immigrant effect” wherein immigrant health on arrival in Canada is better than Canadian-born but declines over time until it is the same or worse than that of Canadian-born [2,3]. This decline is thought to stem from many causes, including poorer access to health care services [4,5]. Access to adequate primary care for this population is important as it is a patient’s first contact with the health care system and a strong primary care system has been shown to reduce health inequities [6].

Access to primary care across the continuum, from initial contact with a provider through to ongoing health care and preventive services, is critical. Previous research has explored the problems immigrants have when first making contact with the primary health care system in Canada and overall, findings show that immigrants are just as likely to have made contact with a family doctor [7-9]. However, certain immigrants groups, such as refugees and those recently arrived, report barriers to receipt of primary care [3,5,7,8]. Common barriers faced by immigrant groups include language difficulties, cultural and societal influences, as well as gender expectations and these factors may affect how easily, and when, immigrants seek care [10,11]. Much less is known about how immigrants fare once they have a primary care provider and how patient, provider and organizational factors may impact access to care.

The goal of this research was first to determine the patient reported access to primary health care services by immigrants who were current users of the primary care system and compare how this differed from the Canadian-born population. The second objective was to explore how the organizational model of primary care might impact access to care for immigrants. We used patient survey data collected for the Comparison on Models of Primary Care (COMPC) study in Ontario, Canada; a practice based study that was designed to identify the impact of organizational features of primary health care on the quality of care across 137 practices in Ontario [12].

## Methods

Data for this study comes from the Comparison of Models of Primary Care (COMPC) Study conducted in Ontario, Canada. The COMPC study used a cross-sectional design where patients, providers and practice administrators were surveyed and chart audits conducted to examine a range of primary care performance parameters. This paper focuses on patient reported access to care and used data that was collected from the patient survey.Data collection took place between October 2005 and June 2006. The study looked at the four principal organizational models of primary care in the province of Ontario at that time. The study was funded by the Ministry of Health and Long-Term Care and was approved by the Ottawa Hospital Research Ethics Board. Full details on the methodology for the entire project can be found in a separate publication and are summarized below [12].

### Primary care practice sample

The unit of sampling and analysis was the primary care practice. We aimed to collect information from 35 practices per model. Practices were required to have worked under their organizational model for at least one year and consent to participate was required from 50% of physicians and nurse practitioners in the practice.

Primary care practices were sampled across Ontario to ensure an equal number of practices within each of the four dominant organizational models at that time; Community Health Centres, Fee-For-Service, Health Service Organizations and Family Health Networks. Community Health Centres have salaried multidisciplinary providers and integral to their mission is the care of an identified, vulnerable population. Fee-For-Service practices remunerate providers based on the number and types of services delivered. The two capitation-based practices (Health Services Organization and Family Health Networks) were considered together, as both were group practices with payment structures predominantly based on a fixed remuneration fee based on the age and sex of enrolled patients.

### Patient population

Participants were included if they were 18 years or older, not severely ill or cognitively impaired, able to communicate in English or French, either directly or through translation, and their provider consented to participate. Following a prepared script, receptionists introduced the study to the patients and handed an invitation letter to all patients presenting for their appointment on the day of survey administration. The survey administrator then provided more detailed information about the study, verified whether the patient met the eligibility criteria, invited eligible patient to participate and obtained informed consent. While the survey was available in English and French only, translation services were offered to the participant. Translation support was provided by staff of the practice, family members or community members. A sequential sample of 30 to 50 patients of each participating providers was identified and eligible participants completed the survey in the waiting room.

### Patient variables

The patient survey captured patient socio-demographic information, including country of birth and date of arrival in Canada. Using country of birth information, immigrants were defined as the non-Canadian-born individuals. The patient survey did not include questions on immigrant status, such as if the respondent was a refugee, asylum seeker, international student. Immigrants were then categorized into four groups denoting number of years since arrival (0-<5years, 5-<10 years, 10-<20 years, ≥20 years), consistent with age categories used in previous research [13]. Immigrants in Canada for less than 5 years were considered “recently arrived”. Age was a variable of specific interest as it naturally increases with time since immigration.

Self-perceived health status was measured on a 5-item Likert scale from poor to excellent and dichotomized to poor (poor, fair) and good (good, very good and excellent) health. Single item measures of self-reported health are strongly predictive of mortality [14] and health service utilization [15] and have been validated in multiethnic populations [16].

### Access measures

The patient survey included questions about the experience of access that were modified from the adult edition of the Primary Care Assessment Tool (PCAT) [16]. Versions of this tool have been used in multi-ethnic and low-income populations [17,18]. Three measures of access, all derived from the patient survey, were used: 1) First Contact Access (four questions from the PCAT that measure access to the source of care during office hours and when the office is closed), and 2) First Contact Utilization (three questions from the PCAT that address the extent to which primary care is the first source of care for various types of health problems) and 3) Number of primary care visits to that practice over the past year (self-reported) (see Table 1). The First Contact Access and First Contact Utilization measures are answered using a 5 item Likert scale (items include: definitely, probably, probably not, definitely not and not sure/don’t remember) [15,16].

**Table 1:** Measures of access to primary care


1	First Contact Accessibility *(all questions answered using a 5 item Likert scale)†
	When your MD/NP’s office is open and you get sick, would someone from this office see you the same day?
	When your MD/NP’s office is open, can you get advice quickly over the phone if you need it?
	When your MD/NP’s office is closed, is there a phone number you can call if you get sick?
	When your MD/NP’s office is closed and you get sick during the night, would someone from this office see you that night?
2	First Contact Utilization *(all questions answered using a 5 item Likert scale †
	When you need a regular general checkup, do you go to your provider before going somewhere else?
	When you have a new health problem, do you go to your provider before going somewhere else?
	When you want to see a specialist, do you get a referral from your provider?
3	Annual health care utilization
	How many visits have you made in the past year to this clinic?

* Adapted from the Primary Care Assessment Tool [19].

† Likert scale items: definitely, probably, probably not, definitely not and not sure/don’t remember.

### Statistical analysis

Patient socioeconomic and demographic profile and self-reported health status were described and compared across immigrant groups. Access scores for the First Contact Access and First Contact Utilization measures were normalized. Multivariate linear and logistic regressions were used to characterize the relationship between access measures and patient and practice characteristics. All analyses were performed using SPSS Version 18. For our analysis we did not adjust for socioeconomic status, as we felt it was an intermediate variable in the causal pathway between immigrant status and outcomes of interest. For the analyses of number of health care visits, we adjusted for self-reported health status and the number of years an individual had been a patient of the practice, as sicker patients and patients new to a practice may use services more frequently than healthy or established patients [20].

## Results

There were 5,361 patients included in the COMPC study, 5,269 (98.3%) reported country of birth and of these 1,099 (20.8%) were immigrants. The profile of immigrants showed variation from Canadian-born across most socioeconomic and demographic variables (Table 2). There was an apparent trend in the immigrant profile with recent immigrants more likely to be younger, non-white and women. The individuals who did not report country of birth had a similar socio-demographic profile to the Canadian-born (not shown).

**Table 2:** Demographic and medical characteristics according to immigrant status and years since migration to Canada (N=5269)

**Characteristics**	**0-<5 years since migration**	**5-<10 years since migration**	**10-<20 years since migration**	**≥ 20 years since migration**	**Canadian-born**
	**n=98**	**n=89**	**n=214**	**n=698**	**n=4170**
**Mean age (years)**	39.8	39.6	39.3	57.9	49.3
**Female (%)**	78.6	77.3	71.0	62.6	66.7
**Education less than high school (%)**	26.7	17.9	13.8	11.9	17.9
**Low Income Cut-off (%)**^**1**^	52.8	43.9	31.4	15.6	14.8
**Home language neither English or French (%)**	23.5	19.1	11.7	3.7	<0.01
**Race non-white (%)**	64.5	71.4	58.4	18.2	2.4
**Rural practice location (%)**^**2**^	2.0	2.2	1.9	5.3	8.8
**Receiving care in a Community health Centre (%)**	69.4	68.5	52.3	15.5	20.3
**Receiving care in a fee-for-service practice (%)**	23	19	29	35	24
**Self reported health good to excellent (%)**	75.2	82.8	84.3	77.7	83.1
**Number of chronic conditions, mean**	1.4	0.9	1.2	2.0	1.8
**Depression reported (%)**	30.6	20.2	27.1	23.8	30.3
**Number of primary care visits in past, mean**	11.5	7.4	7.4	6.3	6.2
**First Contact Access score (normalized %)**	74.0	77.0	74.0	78.0	77.0
**First Contact Utilization score (normalized %)**	94.0	95.0	95.0	98.0	97.0

Percentages reported excludes those with missing data.

^1 ^Low income cut-off according to categorisation by Statistics Canada.

^2 ^Rural Index of Ontario Score >45.

### Health status

After adjustment for age and sex the odds of reporting good health was lower for both recently-arrived immigrants (Odds Ratio (OR) =0.58, 95% Confidence Interval (CI) 0.36, 0.92) and those in Canada for more than 20 years (OR=0.81, 95% CI 0.67, 0.99) compared with Canadian-born individuals (Table 3).

**Table 3:** Unadjusted and adjusted odds ratios (95% CI) of reporting good to excellent health status for immigrant groups by years since arrival compared to the Canadian-born

**Number of years since arrival in Canada**	**Unadjusted**	**Adjusted age and sex**
	**OR (95% CI)**	**OR (95% CI)**
**Non –immigrant Canadian-born (reference)**	1.0	1.0
**0-<5 years**	0.67 (0.42, 1.11)	0.58 (0.36,0.92)*
**5-<10 years**	0.97 (0.57, 1.66)	0.84 (0.49,1.43)
**10-<20 years**	1.10 (0.77, 1.58)	0.93 (0.65, 1.35)
**≥20 years**	0.73 (0.60, 0.88)*	0.81 (0.67, 0.99)*

* Significantly different from Canadian-born population.

### Access to primary health care

Unadjusted scores for First Contact Access and First Contact Utilization were relatively consistent across immigrant categories (Table 2). After adjusting for age and sex, the First Contact Access and First Contact Utilization scores were not significantly different across immigrant groups or compared with Canadian-born (Table 4).

**Table 4:** Adjusted Access and utilization scores of immigrant groups by years since arrival in Canada compared to the Canadian-born adjusted for age, sex, health status and number of years as a patient in the practice

	**First Contact Access**^**1 **^**% difference from Canadian-born (95% CI)**	**First Contact Utilization**^**1 **^**% difference from Canadian-born (95% CI)**	**Number of additional health visits in the past year compared to Canadian-born**^**1 **^**(95% CI)**
**0-<5 years**	0.1 (-3.3, 3.5)	−0.7 (-2.4,0.9)	5.3 (3.5,7.0) *
**5-<10 years**	1.6 (-2.0, 5.2)	−0.7 (-2.4, 0.9)	1.2 (-0.6, 2.9)
**10-<20 years**	−1.6 (-3.8, 0.7)	−0.6 (-1.7,0.5)	1.4 (0.2, 2.6)*
**≥20 years**	−0.2 (-1.6,1.1)	0.3 (-0.4,0.9)	−0.05 (-7.4, 0.6)

^1 ^Adjusted for age, sex, health status and number of years in practice.

* Significantly different from the Canadian-born population.

The number of patient-reported primary care visits in the past year was highest among recent immigrants who made 11.5 visits compared with 6.2 visits among Canadian-born (Table 2). This pattern of health care utilization persisted after adjusting for age, sex, health status and number of years as a patient in the practice. In adjusted analysis recent immigrants had 5.3 (95% CI 3.5, 7.0) more primary care visits in the past year (Table 4) compared to Canadian-born.

While the analysis demonstrated similar First Contact Access and First Contact Utilization between recent immigrants and Canadian-born, a stratified analysis suggested that differences in these measures may exist across primary care models (Table 5). Within Fee-For-Service practices recent immigrants had lower First Contact Access scores compared with Canadian-born. Comparing health care visits, recent immigrants in Fee-For-Service practices had a similar number of health care visits compared with Canadian-born while those recent immigrants in Community Health Centres and capitation practices had more visits compared with Canadian-born.

**Table 5:** Odds ratios of reporting good health and access and utilization for recent immigrants (< 5 years since arrival in Canada) compared to Canadian-born across primary care models

	**Odds of reporting good to excellent health**^**1 **^**OR (95% CI)**	**First Contact Access**^**2 **^**% difference from reference (95% CI)**	**First Contact Utilization**^**2 **^**% difference from reference (95% CI)**	**Number of additional health visits in the past year**^**2 **^**(95% CI)**
**Primary Care Model**				
**Community Health Centre n=68**	0.7 (0.4, 1.3)	2.1 (-2.5,6.7)	0 (-2.5,2.4)	6.2 (2.8,9.6)*
**Fee-For-Service n=23**	0.8 (0.3, 2.1)	−8.0 (-15.0,-10.0)*	−1.4 (-4.5,1.7)	−0.3 (-4.0,3.4)
**Capitation n=7**	1.4 (0.2,12.0)	5.7 (-6.6,18.0)	0 (-2.5,2.4)	4.0 (-0.3,8.2)

^1 ^Adjusted for age and sex.

^2 ^Adjusted for age, sex, health status, number of years as patient in the practice.

* Significantly different from the Canadian-born population.

## Discussion

This study suggests that overall immigrants who make contact with the primary care system experience a similar level of access to, and somewhat higher utilization of, the primary health care system compared with Canadian-born. The rich data collected from this study allowed us to use a number of access measures to explore this relationship and to account for potentially relevant patient and practice factors that might impact access.

We found that recent immigrants reported poorer health compared to the Canadian-born, a finding that was not expected based on the healthy immigrant effect found in population surveys [2,3]. This may be explained by two factors. First, we were unable to distinguish refugees and asylum seekers from other immigrants groups based on the data available. This may have biased our results towards finding a less healthy immigrant population as refugees report poorer health on arrival than other immigrant classes [21]. Second, our study included immigrants who had already accessed primary health care and, thus, may have included less healthy immigrants who required more health care services.

We were encouraged to find that immigrant access to primary care across several measures of access did not differ significantly from Canadian-born. Primary care practice visits were equivalent or higher amongst immigrant groups compared to Canadian-born, after adjusting for factors that relate to need (age, sex, health status) suggesting that immigrants in this population had adequate access to care. Our results agree with previous research that found health care utilization for immigrants varies with years since arrival but over their lifespan immigrants have a similar level of utilization to the Canadian-born [21]. 

The higher use of primary care services reported by recent immigrants in our study can be reasonably explained by a number of factors both medical and socio-demographic [3,21,22]. We see a similar pattern of health care use in other vulnerable populations in Canada where, once initial contact is made, they have more frequent visits [23,24]. Higher utilization for recent immigrants may be driven by initial preventative health care needs (such as vaccinations and health screening) or, given the high proportion of females in this immigrant group, by antenatal or postnatal visits. Socio-demographic variables such as poverty, culture and language may also drive utilization [5,10,25]. Newly arrived immigrants in our study were more likely to be low income, non-white and non-English or French speaking immigrants. Previous research has found that, due to language and cultural communication barriers, immigrants made repeated visits for the same problem because they did not fully understand the care provided in previous visits [22].

Our exploratory analysis of the impact of primary care model identified differences in the distribution of immigrants across models of care and in the access to, and utilization of, care by recent immigrants. More than half of all immigrants in Canada for less than 20 years received care in Community Health Centres; a distribution that may be appropriate given that Community Health Centres have a specific mandate to care for vulnerable populations. In our experience caring for newly arrived immigrants in an urban setting, these patients are often specifically directed to seek care in Community Health Centres. Recent immigrants in Fee-For-Service practices reported poorer access compared with Canadian-born and had fewer primary care visits compared to similar immigrants in other models. The lower performance in two access measures strengthens the likelihood that there may be barriers to access in this model. Recent research from Ontario has suggested that access to primary care services may be different across primary care models for vulnerable populations [26,27]. Future research will need to explore the potential impacts of the organization and structure of primary care on immigrant populations.

### Limitations

The cross-sectional study design means the length of residence categories should be interpreted with caution, as these groups represent different arrival cohorts of immigrants and, thus, changing immigrant populations over time.

We acknowledge that some immigrant groups may have been underrepresented as our population was limited to only those who had accessed primary health care. Furthermore, while translators were available, immigrant patients who did not speak English or French or who had low literacy levels may have been excluded from the study. However, immigrants made up 21% of our study population, a proportion similar to that of the latest national census [1].

We acknowledge that self-reported measures are subject to recall bias and bias of past experience. For immigrants their past experience could have an important impact on their present experience. For example, those who come from countries with very limited access to health care might have lower expectations of care in Canada. We have attempted to minimize these biases by conducting the survey in the waiting room and with the use of access measures that are less subjective and measure patient experience, not simply satisfaction.

## Conclusion

We were encouraged to find that, overall, immigrants who were users of the primary health care system in Ontario, reported similar access to and use of these services compared with the Canadian-born population. While recent immigrants had poorer health status compared with Canadian-born, their higher utilization of primary care services suggests they accessed needed primary care services. We identified some difference in access across primary care models for recent immigrants highlighting the need for continued attention to the role of organizational characteristics of primary health care on access.

## Abbreviations

COMPC: Comparisons of Models Project.

## Competing interests

The authors confirm there are no competing interests.

## Authors’ contributions

EM was responsible for the concept of the research project, the statistical analysis and writing of the manuscript. SD oversaw the research project and statistical analysis, was responsible for the quantitative data collection and participated in the writing of the manuscript. WH conceived the Comparisons of Models of Primary Care study and oversaw its implementation and participated in editing the manuscript. All authors approved the final version to be published.

## Authors’ information

EM is a research fellow at the University of Ottawa, Department of Family Medicine and an early scientist at the C.T. Lamont Primary Health Care Research Centre, University of Ottawa, Ottawa, Ontario, Canada. SD is the director of research operations at the C.T. Lamont Primary Health Care Research Centre, the research arm of the Department of Family Medicine, Faculty of Medicine at the University of Ottawa, Ottawa, Ontario, Canada. WH is a professor at the Department of Family Medicine, the Department of Epidemiology and Community Medicine, and the Institute of Population Health, University of Ottawa, and director of the C.T. Lamont Primary Health Care Research Centre.

This project was supported by funding from the Ministry of Health and Long Term Care.

## Pre-publication history

The pre-publication history for this paper can be accessed here:

http://www.biomedcentral.com/1471-2296/13/128/prepub

## References

[B1] Chui T Tran K Maheaux H Immigration in Canada: a portrait of the foriegn-born population, 2006 census Statistics Canada 2006 Census: Findings 2007 2010

[B2] Newbold B Health status and health care of immigrants in Canada: a longitudinal analysis J Health Serv Res Policy 2005 10 2 78 83 10.1258/1355819053559074 15831190

[B3] McDonald J Kennedy S Insights into the healthy immigrant effect: health status and health service use of immigrants to Canada Soc Sci Med 2004 59 1613 27 10.1016/j.socscimed.2004.02.004 15279920

[B4] Newbold B Health care use and the Canadian immigrant population Int J Health Serv 2009 39 3 545 65 10.2190/HS.39.3.g 19771955

[B5] Miedema B Hamilton R Easley J Climbing the walls: structural barriers to accessing primary care for refugee newcomers in Canada CMAJ 2008 54 3 335 6 PMC2278339 18337519

[B6] Shi L Starfield B Politzer R Regan J Primary care, self-rated health, and reductions in social disaprities in health Health Serv Res 2002 37 3 529 50 10.1111/1475-6773.t01-1-00036 12132594 PMC1434650

[B7] Setia M Access to health-care in Canadian Immigrants: a longitudinal study of the National Population Health Survey Health and Soc Care 2011 19 1 70 79 10.1111/j.1365-2524.2010.00950.x PMC3762743 21054621

[B8] Glazier R Creatore M Cortinois A Agha M Moineddin R Neighbourhood recent immigration and hospitalization in Toronto, Canada Can J Public Health 2004 95 3 130 4 10.1007/BF03403663 PMC6975793 15191130

[B9] LeBrun L Dubay L Access to primary and preventative care among foreign-born adults in Canada and the United states Health Serv Res 2010 1 45 1693 719 20819107 10.1111/j.1475-6773.2010.01163.x PMC3026954

[B10] Pottie K Ng E Spitzer D Mohammed A Glazier R Language proficiency, gender, and self-reported health: an analysis of the first two waves of the longitudinal survey of immigrants to Canada Can J Public Health 2008 99 6 505 10 19149396 10.1007/BF03403786 PMC6975753

[B11] Gushulak BD Pottie K Hatcher Roberts J Torres S DesMeules M Migration and health in Canada: health in the global village CMAJ 2011 183 12 E952 E958 20584934 10.1503/cmaj.090287 PMC3168671

[B12] Dahrouge S Hogg W Russell G Geneau R Kristjansson E Muldoon L The comparison of models of primary care in Ontario (COMP-PC) study: methodology of a multifaceted cross-sectional practice-based study Open Med 2009 3 3 e149 e164 21603051 PMC3090123

[B13] Ali J McDermott S Gravel R Recent research on immigrant health from statistics Canada’s population surveys Can J Public Health 2004 95 3 9 13 10.1007/BF03403659 PMC6975564 15191126

[B14] Idler E Benyamini Y Self-rated health and mortality: a review of twenty-seven community studies J Health Soc Behav 1997 38 1 21 37 10.2307/2955359 9097506

[B15] DeSalvo K Fan V McDonell M Fihn S Predicting mortality and healthcare utlization with a single question Health Serv Res 2005 40 4 1234 46 10.1111/j.1475-6773.2005.00404.x 16033502 PMC1361190

[B16] Shi L Starfield B Xu J Validating the adult primary care assessment tool J Fam Pract 2001 50 2 161

[B17] Lee J Choi Y Sung N Kim S Chung S Kim J Development of the Korean primary care assessment tool–measuring user experience: tests of data quality and measurement performance Int J Qual Health Care 2009 21 103 11 10.1093/intqhc/mzp007 19286829

[B18] Harzheim E Duncan B Stein A Cunha C Goncalves M Trindade T Quality and effectiveness of different approaches to primary care delivery in Brazil BMC Health Serv Res 2006 6 156 10.1186/1472-6963-6-156 17147819 PMC1790713

[B19] Kobayashi K Prus S Lin Z Ethnic differences in self-rated and functional health: does immigrant status matter? Ethn Health 2008 13 2 129 47 10.1080/13557850701830299 18425711

[B20] Jabaaij L De Bakker D Schers H Bindels P Dekker J Schellevis F Recently enlisted patients in general practice use more health care resources BMC Fam Pract 2007 1 8(64) 10.1186/1471-2296-8-64 PMC2235863 18047642

[B21] Newbold B The short term health of Canada’s new immigrant arrivals: evidence from LSIC Ethn Health 2009 14 3 315 36 10.1080/13557850802609956 19263262

[B22] Leduc N Proulx M Patterns of health services utilization by recent immigrants J Immigr Health 2004 6 1 15 27 14762321 10.1023/B:JOIH.0000014639.49245.cc

[B23] Asada Y Kepart G Equity in health services use and intensity of use in Canada BMC Health Serv Res 2007 7 41 10.1186/1472-6963-7-41 PMC1829158 17349059

[B24] Glazier R Tepper J Agha MM Jaakkimainen L, Upshur R, Klein-Geltink J Primary Care in Disadvantaged Populations 2006 Toronto, Ontario: Institute for Clinical Evaluative Sciences. Primary Care in Ontario: ICES Atlas 121 140

[B25] Carrasco C Gillespie M Goodluck M Accessing primary care in Canada: giving voice to the perceptions and experiences of racialized immigrants (a systematic review) 2009

[B26] Glazier R Klein-Geltink J Kopp A Sibley L Capitation and enhanced fee-for-service models for primary care reform: a population-based evaluation CMAJ 2009 180 11 E72 E81 19468106 10.1503/cmaj.081316 PMC2683211

[B27] Glazier R Zagorski B Rayner J Comparison of primary care models in Ontario by demographics, case mix and emergency department Use 2008/09 to 2009/10: ICES investigative report 2012

